# Persistent Magnetism and Tunable Doping of Monolayer Graphene via Europium Density Modulation

**DOI:** 10.1002/advs.202521592

**Published:** 2025-11-21

**Authors:** M. Jugovac, I. Cojocariu, G. Bihlmayer, P. Gargiani, M. Valvidares, C. A. Brondin, S. Blügel, A. Locatelli, T. O. Mentes, P. Perna

**Affiliations:** ^1^ Dipartimento di Fisica Università degli studi di Trieste Via A. Valerio 2 Trieste 34127 Italy; ^2^ Elettra Sincrotrone Trieste S.C.p.A. Strada statale 14, km 163.5 Trieste 34149 Italy; ^3^ Peter Grünberg Institut (PGI‐1) Forschungszentrum Jülich and JARA 52425 Jülich Germany; ^4^ ALBA Synchrotron Light Source Carrer de la Llum 2‐26 Barcelona 08290 Spain; ^5^ CNR‐Istituto di Struttura della Materia (CNR‐ISM) Strada Statale 14, km 163.5 Trieste 34149 Italy; ^6^ Institute for Theoretical Physics RWTH Aachen University 52056 Aachen Germany; ^7^ IMDEA Nanociencia C/ Faraday 9, Campus de Cantoblanco Madrid 28049 Spain

**Keywords:** doping, flat bands, graphene, interfacial magnetism, nanotechnology

## Abstract

Reaching the van Hove singularity (VHS) in a material enables the emergence of exotic electronic and magnetic phases, such as superconductivity and the quantum anomalous Hall effect. This is demonstrated in cuprates, magic‐angle bilayer graphene, and more recently, monolayer graphene interfaced with alkali and rare earth elements. Here, the europium density at the graphene/rhenium interface is modulated to tune the electron doping level in monolayer graphene across the VHS point, forming either a dense or diluted europium phase. The dense phase enables flat bands at the Fermi level, while graphene remains decoupled from the Re(0001) substrate in both cases. The Dirac point is shifted over 1.5 eV below the Fermi level, and europium lifts the degeneracy of the Dirac cones: one branch hybridizes with Eu 4f states, the other retains Dirac‐like dispersion, as corroborated by density functional theory. X‐ray absorption spectroscopy reveals a mixed Eu(II)/Eu(III) valence state in the dense phase and the persistence of Eu magnetic response up to room temperature in both. The intercalated phases exhibit exceptional thermal stability, with the diluted phase stable up to 960 K. These results highlight the potential of rare‐earth‐doped graphene for engineering flat bands, tunable Dirac‐cone splitting, and robust interfacial magnetism.

## Graphical abstract

The modulation of europium density at the graphene/rhenium interface enables electron doping of monolayer graphene both beneath and beyond the van Hove singularity. The interfacial europium is ferromagnetic, with a transition from a mixed Eu(II)/Eu(III) valence in the dense phase to a pure Eu(II) state in the diluted phase. Strong Re‐Eu interaction ensures exceptional thermal stability and potential applications.

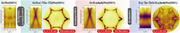

## 1 Introduction

Graphene, with its exceptional electronic, mechanical, and thermal properties, is a focal point in materials science and nanotechnology. These outstanding characteristics make it a highly sought‐after material for various applications ranging from flexible electronics to advanced quantum devices. One of the most compelling aspects of graphene research is doping,^[^ ^1^ ^]^ which involves the introduction of impurities or the interaction with other materials to modulate its electronic properties.^[^ ^2^, ^3^, ^4^, ^5^, ^6^, ^7^ ^]^ This approach is essential for tailoring graphene's intrinsic properties and expanding its technological potential.^[^ ^8^, ^9^ ^]^ Electron doping, achieved by interfacing graphene with alkali metals or rare earth elements, can significantly impact its conductivity and band structure.^[^ ^10^ ^]^ This doping method stands out for its ability to provide reversible and controllable modifications of graphene's electronic properties, enabling precise tuning for specific functionalities. We note that this approach is remarkably different from the case of magic‐angle‐bilayer graphene, where flat‐band physics arises from moiré superlattices and twist‐angle control rather than charge transfer.^[^ ^14^, ^15^ ^]^

Due to their substantial magnetic moment, short spin relaxation times, and strong spin‐orbit coupling (SOC) relative to the crystal field, 4f‐elements are compelling dopants for imparting novel functionalities to graphene.^[^ ^16^, ^17^, ^18^, ^19^ ^]^ In this context, europium's capacity to induce significant alterations in graphene's electronic properties positions it as an excellent candidate for the advancement of graphene‐based technologies.^[^ ^20^ ^]^ Importantly, driving graphene toward a high density of states at the Fermi level — approaching or even crossing the van Hove singularity (VHS) — has been shown to favor interaction‐driven phases and to stabilize otherwise inaccessible electronic and magnetic states, including flat dispersions near the Fermi level in Eu‐intercalated graphene on magnetic substrates.^[^ ^12^, ^13^ ^]^ Such regimes are of strong interest because enhanced many‐body interactions can promote correlated electronic behavior and nontrivial magnetic responses.^[^ ^21^, ^22^, ^23^ ^]^

Besides alkali and rare earth doping, the choice of the substrate plays a critical role in modulating the doping level and overall performance of graphene. In particular, the nature and symmetry of the substrate determine whether graphene is weakly or strongly bound to it.^[^ ^24^, ^25^ ^]^ In the case of graphene/Re(0001), the strong covalent interaction occurring at the interface is closely related to the hybridization between C 2*p_z_* orbital and Re $5d_{z^{2}}$ orbital, which is absent in other weak van der Waals interacting interfaces, such as graphene/Ir(111).^[^ ^26^ ^]^ Additionally, the graphene layer is strongly buckled with regions where the carbon atoms are close to the Re ones and regions where they lie much farther.^[^ ^27^ ^]^ The strong coupling at the interface leads to significant modifications in graphene's electronic band structure, such as the opening of a wide band gap of almost 4 eV.^[^ ^28^ ^]^

Previous studies have shown that intercalation of rare earth elements under graphene typically results in the presence of a $\left(\sqrt{3}\times \sqrt{3}\right)R30^{\circ }$ reconstruction, which reflects a hexagonal planar arrangement commensurate with graphene, i.e., EuC_6_,^[^ ^19^ ^]^ along with the flattening of the π^*^ band and the approaching of the VHS regime.^[^ ^11^, ^12^, ^29^, ^30^ ^]^ However, when europium is intercalated at the graphene/Re(0001) interface, we observe a distinct behavior. Instead of the conventional $\left(\sqrt{3}\times \sqrt{3}\right)R30^{\circ }$ phase, two europium superstructures are stabilized: a high‐density $\left(\sqrt{13}\times \sqrt{13}\right)R13.9^{\circ }$ phase and a lower‐density (4 × 4) phase. These phases can be reversibly accessed by thermal treatment and are exceptionally stable, with the diluted europium phase remaining intact up to ≈960 K. By tuning the europium density between these two phases, the position of the graphene Dirac point can be shifted by more than 1.5 eV below the Fermi level, allowing us to populate the π^*^ band up to the van Hove singularity and to generate flat bands at the Fermi level in the europium‐dense phase. Upon reducing the europium density, the overall electron doping decreases and the flat portion of the π band moves above the Fermi level, but the intercalated layer remains structurally and magnetically robust.

Magnetic characterization shows strong ferromagnetic behavior at low temperature in both intercalated systems, with a dominant Eu(II) character at the M_5_,_4_ edge, and a finite dichroic response still detectable at room temperature under an applied field. The $\left(\sqrt{13}\times \sqrt{13}\right)R13.9^{\circ }$ phase exhibits a higher remanent magnetization than the (4 × 4) phase, reflecting the higher europium density.

Spin‐resolved DFT calculations further reveal that europium at the graphene/Re(0001) interface does not simply open a full gap at the Dirac point, as observed for europium intercalated on Co(0001) where a $\left(\sqrt{3}\times \sqrt{3}\right)R30^{\circ }$ superstructure folds $\bar{K}$ and ${\bar{K}}^{\prime }$ and enforces intervalley mixing. Instead, in the present $\left(\sqrt{13}\times \sqrt{13}\right)R13.9^{\circ }$ and (4 × 4) phases on Re(0001), the europium superstructures do not superpose K and K′ in the reduced Brillouin zone. As a consequence, graphene does not develop a full Dirac‐point gap; rather, the Dirac cones become strongly split, with one branch hybridizing with Eu 4f states and shifting in energy while the other branch remains largely intact and preserves a linear dispersion near K. This establishes graphene/Eu/Re(0001) as a platform where extreme electron doping, access to flat bands at the Fermi level, tunable Dirac‐cone splitting without complete gap opening, and persistent interfacial magnetism can all be controlled through the europium density at the interface.

## 2 Results and Discussion

**Figure 1:**
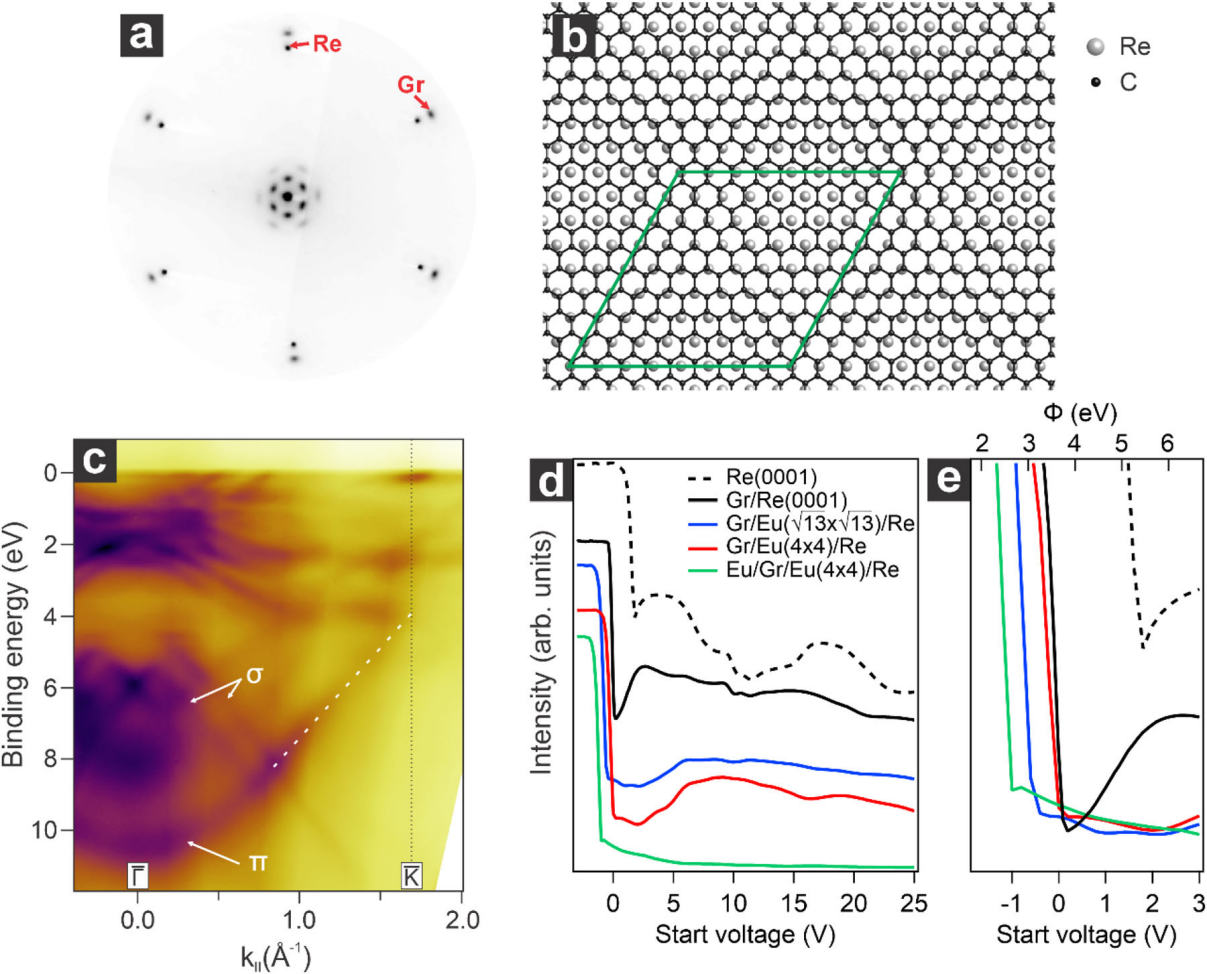
Structural and electronic properties of the graphene/Re(0001) interface. a) Corresponding LEED pattern (E_k_ = 70 eV) and b) structural atomic model showing the Gr(10x10)/Re(9x9) with the relative unit cell in green. c) Energy versus momentum map of Gr/Re(0001) acquired along the $\bar{\Gamma K}$ direction of the surface Brillouin zone. d) Comparison of LEEM‐IV curves of Gr/Re with the other ones relative to the different preparations showcased below. e) Zoom in on the low‐energy region of the spectra in d).

Graphene growth on Re(0001) is performed via chemical vapor deposition (CVD) by exposing the hot catalytic surface to ethylene. In particular, the clean rhenium surface is exposed to ethylene at room temperature, followed by annealing cycles between 570 and 970 K, based on the well‐known growth recipe.^[^ ^31^ ^]^ Such a procedure allows for obtaining monolayer graphene, with only a small amount of carbides, whose formation is otherwise favored on the rhenium surface. Despite the significant lattice mismatch between graphene (2.465 Å) and rhenium (2.761 Å), graphene grows with its crystalline axis aligned with the underlying substrate, as verified by LEED (**Figure** **1**a). Due to such lattice mismatch, graphene forms an incommensurate phase on Re(0001) that exhibits a moiré pattern consistent with a (10 × 10)‐graphene unit cell over a (9 × 9)‐Re(0001) unit cell (Figure 1b).^[^ ^31^ ^]^ This moiré pattern is clearly visible near the specular LEED reflection. The close contact at the interface induces a substantial buckling of the graphene layer^[^ ^26^ ^]^ and a strong orbital hybridization.^[^ ^28^ ^]^ Such interaction broadens the graphene‐related bands; hence, the Dirac cone's apex is not directly visible in the corresponding ARPES spectrum (Figure 1c). However, by fitting the linear part of the π band, the Dirac point is found at 3.92 eV, in agreement with previous reports.^[^ ^28^ ^]^ Graphene adsorption on Re also alters electron reflectivity in the low‐energy region (Figure 1d), increasing the intensity between 5 and 15 V compared to the bare Re substrate (black vs black dashed curve), compatible with the presence of a graphitic layer. Moreover, the onset of the mirror electron microscopy to low energy electron microscopy (MEM‐LEEM) transition (Figure 1e), whose energy position is closely related to work function changes, shifts upon graphene adsorption by 1.5 V toward lower energies, consistent with previous reports on similar systems.^[^ ^32^ ^]^

**Figure 2:**
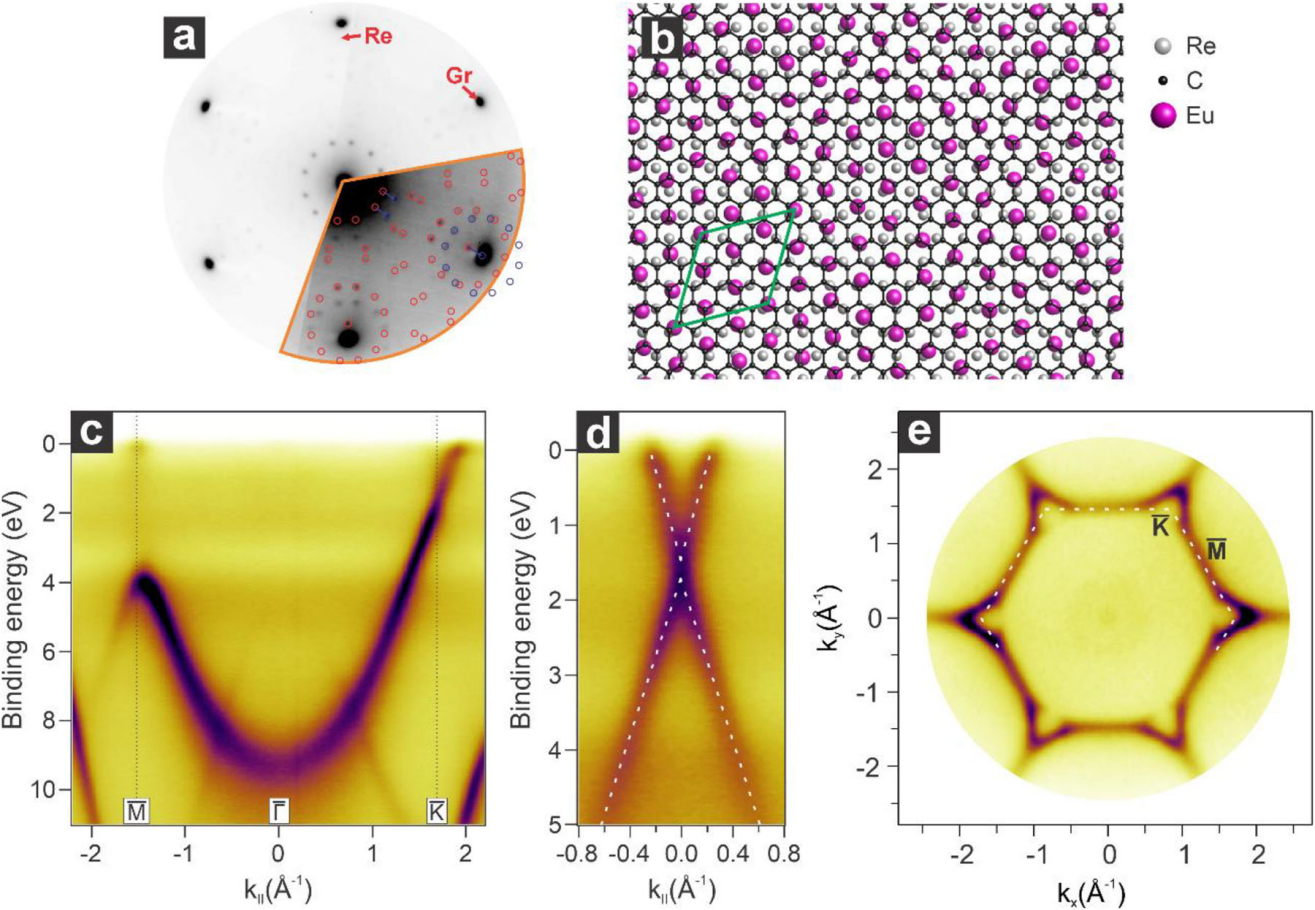
Structural and electronic properties of the graphene/Eu $\left(\sqrt{13}\times \sqrt{13}\right)R13.9^{\circ }$ /Re(0001) interface. a) Corresponding LEED pattern (E_k_ = 70 eV) along with the simulated model indicated with red circles and the shifted ones in blue. b) Structural atomic model of the interface along with the unit cell indicated in green. c) Energy versus momentum map acquired along the $\bar{M\Gamma K}$ direction and d) at the $\bar{K}$ point of the SBZ. e) Fermi surface of the interface along with the SBZ indicated by the white dashed line.

On top of Gr/Re(0001), metallic europium is deposited while keeping the sample at 470 K. This deposition results in the $\left(\sqrt{3}\times \sqrt{3}\right)R30^{\circ }$ reconstruction (see Figure S1a, Supporting Information), commonly observed when rare earth elements are deposited on graphene,^[^ ^12^, ^29^, ^33^ ^]^ which reflects an Eu hexagonal planar arrangement commensurate with graphene, i.e., EuC_6_. The Eu evaporation is stopped after the $\left(\sqrt{3}\times \sqrt{3}\right)R30^{\circ }$ reconstruction reaches saturation. In order to intercalate Eu at the Gr/Re interface, the sample is annealed while monitoring the changes in LEED (see Figure S1, Supporting Information). During annealing, the crystalline order of the $\left(\sqrt{3}\times \sqrt{3}\right)R30^{\circ }$ reconstruction improves until a temperature of 630 K is reached. Beyond this temperature, Eu atoms begin to intercalate at the Gr/Re interface, with the process completing ≈650 K. The resulting LEED pattern presents major changes, especially in the proximity of the zero‐ and first‐order diffraction spots. Indeed, the Gr/Re moiré spots disappear, and 12 additional spots become visible around each of the seven main diffraction spots (**Figure** **2**a). Modeling the LEED pattern reveals a $\left(\sqrt{13}\times \sqrt{13}\right)R13.9^{\circ }$ superstructure relative to the Re(0001) surface (Figure 2b), with an Eu‐Eu nearest neighbor distance of 3.77 Å. However, additional spots in the LEED pattern, not part of the primary superstructure, are also observed. These extra spots, marked by blue circles in Figure 2a, can be explained as arising from double scattering between the graphene and Re principal crystal vectors.^[^ ^34^ ^]^ Analysis of the surface unit cell area provides insights into the carbon to europium ratio, which is determined to be Eu:C = 1:4.59. This value is much lower than the reported ratio for the stable EuC_6_ phase, which is characteristic of the $\sqrt{3}\times \sqrt{3}R30^{\circ }$ Gr/Eu interface.^[^ ^12^, ^33^ ^]^ This finding indicates that the rhenium substrate plays a crucial role in stabilizing a dense interfacial europium phase, enabling higher doping levels in graphene. Additionally, the intercalation of Eu at the interface significantly lowers the system's work function. Compared to the Gr/Re system, the work function decreases by 0.8 eV following Eu intercalation, as shown in Figure 1e.

Angle‐resolved photoemission spectroscopy (ARPES) reveals substantial changes in the electronic structure following Eu intercalation as compared to the pristine Gr/Re interface. As visible in Figure 2c, the spectrum acquired along the $\bar{M\Gamma K}$ direction is dominated by a sharp and well‐defined π band. This indicates that the presence of interfacial Eu decouples graphene from the substrate and lifts the interfacial hybridization. Additionally, the charge transfer from the intercalated Eu atoms to the graphene layer results in the occupation of the π* band, shifting the Dirac point to a binding energy of 1.61 eV and opening a 0.21 eV bandgap (Figure 2d), which is located in close proximity of the Eu 4f states. This charge transfer is sufficient to populate the π* band up to the VHS, as manifested by the appearance of flat bands (Figure 2e) at the $\bar{M}$ points of the surface Brillouin zone (SBZ). The Fermi velocity, determined by fitting the linear part of the π band in Figure 2d, is v_F_ = 0.88 × 10^6^ m/s, consistent with the value obtained for Eu‐intercalated Gr/Co.^[^ ^12^ ^]^ Furthermore, following Luttinger's theorem,^[^ ^38^ ^]^ the calculated electron carrier density is n = 2.46 × 10^14^ cm^−2^.

Compared to other graphene/rare earth systems, such as Gr/Eu/Co,^[^ ^12^ ^]^ Gr/Eu/Ni,^[^ ^35^ ^]^ Gr/Yb/SiC,^[^ ^29^ ^]^ the doping level observed in this system is among the highest ever observed. This enhanced doping can be attributed to the hybridization between Eu and Re, which amplifies the electron‐doping effect of the rare earth element alone, potentially resulting in a stronger Rashba effect at the interface.

**Figure 3:**
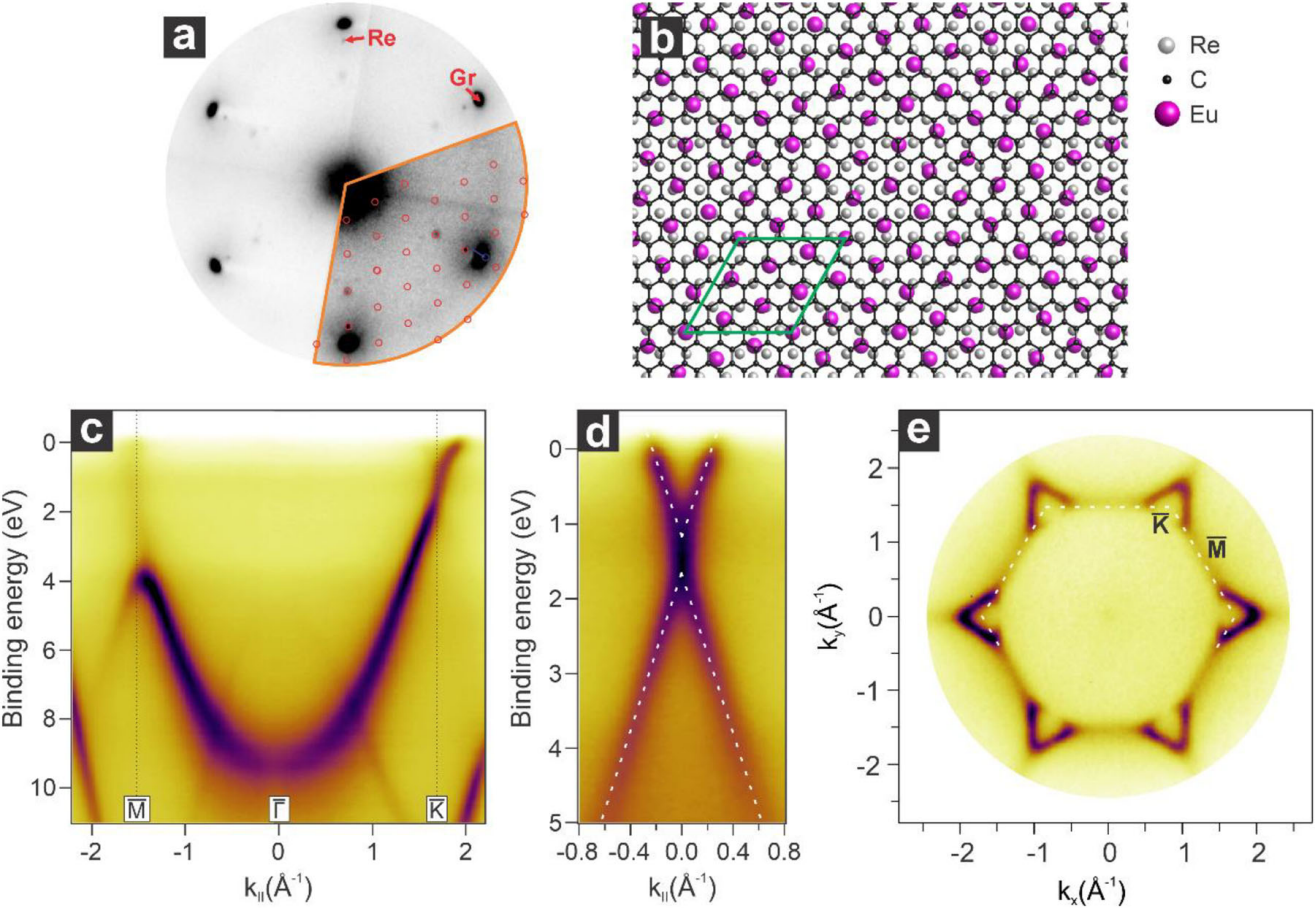
Structural and electronic properties of the graphene/Eu(4 × 4)/Re(0001) interface. a) Corresponding LEED pattern (E_k_ = 70 eV) along with the simulated model indicated with red circles. b) Structural atomic model of the interface along with the unit cell indicated in green. c) Energy versus momentum map acquired along the $\bar{M\Gamma K}$ direction and d) at the $\bar{K}$ point of the SBZ. e) Fermi surface of the interface along with the SBZ indicated by the white dashed line.

The $\left(\sqrt{13}\times \sqrt{13}\right)R13.9^{\circ }$ phase is thermally stable up to ≈900 K. If this temperature is exceeded, a structural transformation takes place at the interface. After annealing the sample to 960 K, the LEED pattern shows sharp and intense graphene (Gr) and rhenium (Re) spots, along with six additional spots aligned with the high‐symmetry directions of Gr and Re. These spots are attributed to a (4 × 4) superstructure of the Eu atoms on Re (**Figure** **3**b), with an Eu‐Eu nearest neighbor distance of 4.19 Å. This new unit cell is 23% bigger than the previous one, resulting in a Eu:C ratio of 1:5.64, closer in stoichiometry to the EuC_6_ phase. The reduction in Eu coverage from the $\left(\sqrt{13}\times \sqrt{13}\right)R13.9^{\circ }$ to the (4 × 4) structure could be attributed to the segregation of Eu into the rhenium bulk or to deintercalation followed by evaporation. The lower Eu concentration at the interface also affects the system's work function, increasing it by 0.55 eV compared to the previous intercalation case.

The reduced Eu coverage also impacts the electronic structure of the Gr/Eu/Re system. The energy versus momentum map acquired along the $\bar{M\Gamma K}$ direction is characterized by the presence of a single graphene parabolic band (Figure 3c), similar to the case of the graphene/Eu $\left(\sqrt{13}\times \sqrt{13}\right)R13.9^{\circ }$ phase. However, the lower doping level shifts the graphene Dirac point to a binding energy of 1.40 eV (Figure 3d). As a result, the VHS regime is no longer reached, and the flat portion of the π* band moves into the unoccupied energy spectrum. The 2D momentum map at the Fermi level (Figure 3e) shows only electron pockets at the $\bar{K}$ point of the surface Brillouin zone, and almost zero intensity at the $\bar{M}$ point. This resembles the case of Li‐intercalated graphene, where the photoemission signal around the $\bar{M}$ point was demonstrated to originate from the spectral tail of the unoccupied part of the π* band located above the Fermi level.^[^ ^11^ ^]^ The lower level of doping corresponds to a reduced electron carrier density as compared to the graphene/Eu $\left(\sqrt{13}\times \sqrt{13}\right)R13.9^{\circ }$ /Re case, being n = 2.24 × 10^14^ cm^−2^. On the other hand, while being less electron‐doped, the increased level of hybridization reflects in a lowering of the Fermi velocity (v_F_ = 0.80 × 10^6^ m/s), i.e., demonstrating a stronger interaction of graphene with respect to the interface.

Interestingly, as compared to the previous case, the bandgap at the Dirac point increases to 0.47 eV. This can be explained by examining the energy‐momentum maps of the bare Eu/Re interface (Figure S2, Supporting Information), obtained by depositing Eu on clean Re and annealing to 650 and 960 K. In the case of the Eu $\left(\sqrt{13}\times \sqrt{13}\right)R13.9^{\circ }$ /Re interface, the center of the Eu 4f band is found at 1.18 eV. Instead, in the case of Eu(4 × 4)/Re, the Eu 4f band is centered at 1.39 eV, in correspondence with the graphene Dirac point. As observed in previous works,^[^ ^12^, ^13^ ^]^ the increased bandgap opening occurs due to the strong hybridization between the graphene π‐band and the Eu 4f band when the energy levels of the 4f band and the Dirac point coincide. In this respect, Rosenzweig et al. (graphene/Yb/SiC) showed that annealing reduces Yb coverage, shifts the Dirac point toward the Fermi level (lower n‐doping), and drives the Yb 4f doublet to have an increased binding energy.^[^ ^29^ ^]^ Analogously, in our system, the transition from the dense to dilute Eu phase reduces doping and shifts the Eu 4f spectral weight to higher binding energy, consistent with lower electron density and enhanced hybridization of the intercalant with the Re substrate.

**Figure 4:**
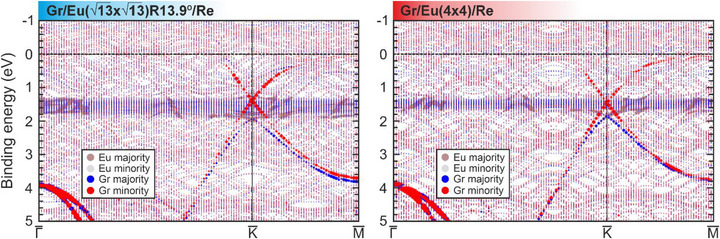
Density functional theory band structures calculated along the high‐symmetry directions for (left) Gr/Eu $\left(\sqrt{13}\times \sqrt{13}\right)R13.9^{\circ }$ /Re(0001) and (right) Gr/Eu(4 × 4)/Re(0001) heterostructures.

To further rationalize the experimental findings, we performed DFT calculations for both the $\left(\sqrt{13}\times \sqrt{13}\right)R13.9^{\circ }$ and (4 × 4) europium superstructures intercalated at the graphene/Re(0001) interface (Figure 4). Details about the optimized structure are found in Figure S3 (Supporting Information), and the structural optimization is described in the Methods section. In the case of the $\left(\sqrt{13}\times \sqrt{13}\right)R13.9^{\circ }$ intercalated structure, the graphene Dirac point is found at 1.55 eV below the computed Fermi level with the presence of a spin‐splitting of the Dirac cone of ≈0.6 eV. The strong intensity of Eu states above the majority spin cone makes this splitting appear smaller in experiment. Moreover, the strong interfacial hybridization induces a clear spin‐splitting along the whole graphene π band. In the case of the (4 × 4) intercalated superstructure, the graphene Dirac point is found at 1.50 eV below the computed Fermi level, with the opening of a spin‐splitting of the Dirac cone of 0.4 eV. Note the smaller 4*f* band‐width due to the lower density of Eu atoms. The induced net spin polarization in graphene is small, governed mainly by the spin‐split states in the proximity of the Fermi level, which show a single spin character. In the case of the Eu (4 × 4) superstructure, the spin polarization in graphene amounts to −0.007 µ_B_ per C atom on average (all negative), while, for the $\left(\sqrt{13}\times \sqrt{13}\right)R13.9^{\circ }$ superstructure, the carbon atoms show mixed signs with an average magnitude of ≈0.004 µ_B_.

It is instructive to compare these results with those obtained for graphene/Eu/Co(0001).^[^ ^12^ ^]^ In that case, the $\left(\sqrt{3}\times \sqrt{3}\right)R30^{\circ }$ periodicity imposed by europium causes $\bar{K}$ and ${\bar{K}}^{\prime }$ to overlap in the reduced Brillouin zone; this superposition enables intervalley mixing and opens a bandgap at the Dirac point. On the other hand, in the graphene/Eu/Re(0001) system investigated here, the situation is fundamentally different: for both the $\left(\sqrt{13}\times \sqrt{13}\right)R13.9^{\circ }$ and (4 × 4) europium superstructures, the periodicity does not superpose $\bar{K}$ and ${\bar{K}}^{\prime }$ in the reduced Brillouin zone. Instead, it lifts the degeneracy between the two spin branches of the Dirac cone: the majority branch hybridizes with the Eu 4f states and shifts toward higher binding energies, while the minority one remains largely unperturbed and maintains a linear dispersion at $\bar{K}$. As a result, a conducting Dirac‐like channel is preserved while, at the same time, the two branches are no longer equivalent in energy. By switching between the dense $\left(\sqrt{13}\times \sqrt{13}\right)R13.9^{\circ }$ phase and the diluted (4 × 4) phase, the hybridization strength and the separation between the two branches can be tuned thermally. In practice, this means that europium on Re(0001) does not simply gap graphene, but produces a regime in which one branch of the Dirac cone is selectively coupled to Eu 4f states and the other remains available near the Fermi level.

**Figure 5:**
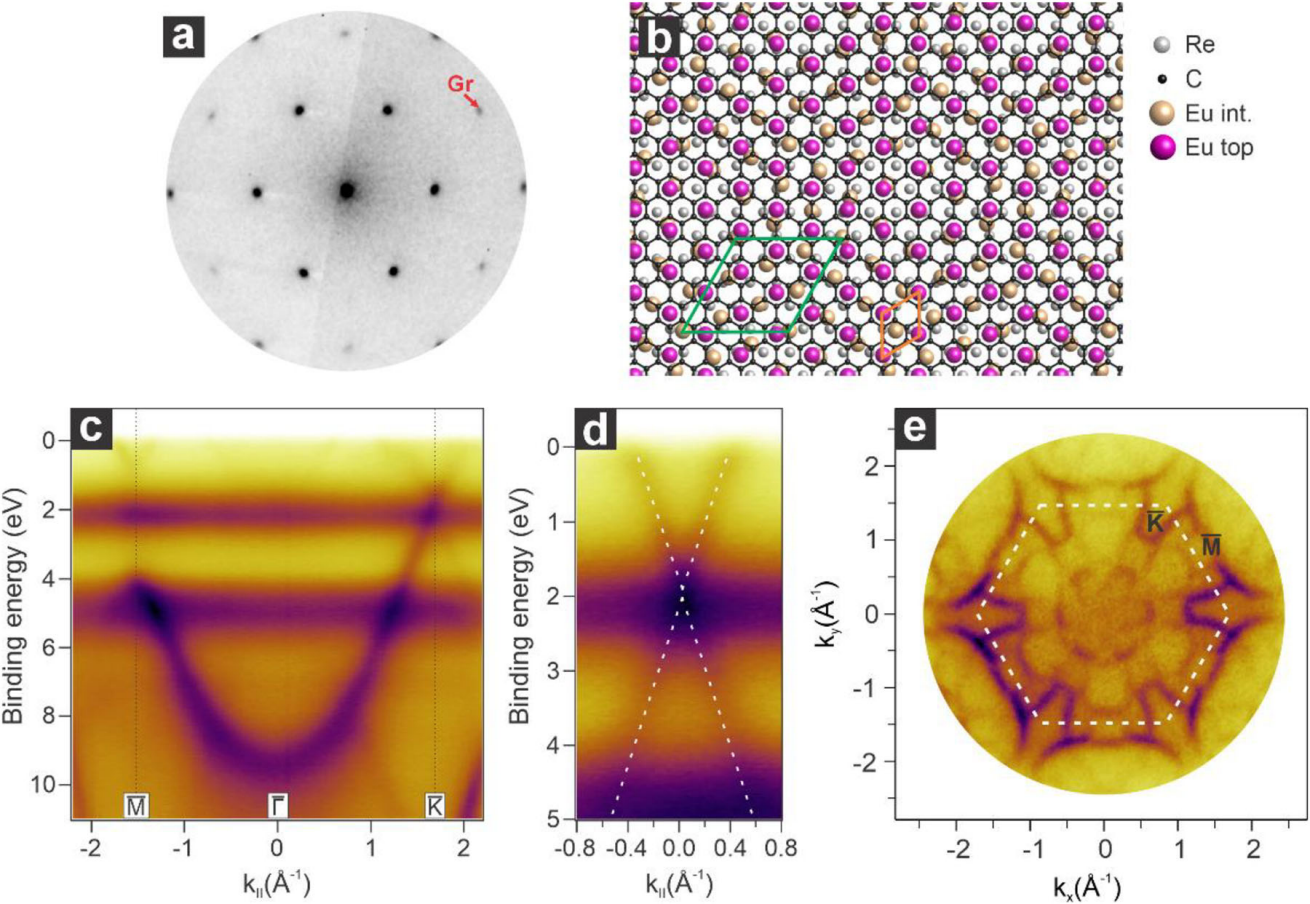
Structural and electronic properties of the Eu($\sqrt{3}\times \sqrt{3}$)/graphene/Eu(4 × 4)/Re(0001) interface. a) Corresponding LEED pattern (E_k_ = 70 eV). b) Structural atomic model of the interface along with the unit cell indicated in green. c) Energy versus momentum map acquired along the $\bar{M\Gamma K}$ direction and d) at the $\bar{K}$ point of the SBZ. e) Fermi surface of the interface along with the SBZ indicated by the white dashed line.

The electronic properties of the system can be further modified by depositing additional Eu on top of the Gr/Eu(4 × 4)/Re(0001) interface while maintaining the sample at 470 K. At this temperature, Eu accumulates on top of the graphene layer without intercalating at the interface. The deposition was halted upon reaching a saturated $\left(\sqrt{3}\times \sqrt{3}\right)R30^{\circ }$ reconstruction on top of the graphene (see LEED pattern in **Figure** **5**a). This additional Eu deposition causes a work function decrease of 0.97 eV compared to the Gr/Eu(4 × 4)/Re(0001) interface, reaching a value of 2.43 eV (see Figure 1e).

The energy versus momentum map acquired along the $\bar{M\Gamma K}$ direction is characterized by the presence of the graphene parabolic band along with two intense non‐dispersive contributions at ≈2 and 5 eV. The lower‐energy feature is attributed to emission from the Eu 4f state, while the higher‐energy feature corresponds to emission from europium 5d and 6s states.^[^ ^36^ ^]^ These contributions are also present in the two intercalated systems discussed before, with a weaker intensity mainly due to the photoelectron screening by graphene. The 1:1 ratio observed between the Eu 4f band and the 5d/6s peak characterizes metallic europium in this range of photon energies.^[^ ^41^ ^]^ Enhanced charge transfer from the top Eu layer causes a further downward shift of the Dirac point, now located at 1.91 eV (Figure 5d). Interestingly, the bandgap narrows to just a few tens of meV. This behavior can be explained by the antiferromagnetic coupling between the two Eu layers, as demonstrated in a recent study.^[^ ^13^ ^]^ This coupling compensates for the spin‐dependent gap opening observed in the intercalated system, effectively reducing it to zero. The electron carrier density in this configuration is estimated at n = 5.74 × 10^14^ cm^−2^, one of the highest values ever reported for substrate‐supported graphene.

The presence of the $\left(\sqrt{3}\times \sqrt{3}\right)$ R30° reconstruction on top of the graphene also induces Brillouin zone folding. As shown in Figure 5e, the Fermi surface of the system features six electron pockets near the $\bar{K}$ points of the surface Brillouin zone (SBZ), along with intensity around the $\bar{\Gamma }$ point due to the folding effect, consistent with observations in similar systems.

**Figure 6:**
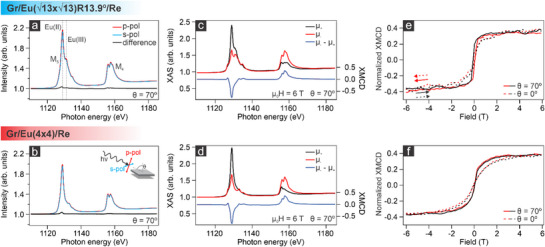
Magnetic properties of the graphene/Eu $\left(\sqrt{13}\times \sqrt{13}\right)R13.9^{\circ }$ /Re(0001) and graphene/Eu(4 × 4)/Re(0001) interfaces. a,b) XAS acquired at the Eu M_4,5_ edge for the two phases in grazing incidence geometry (T = 300 K). c,d) Absorption spectra acquired with left and circular polarized light and the corresponding difference (T = 4 K, µ_0_H = 6 T). e,f) XMCD magnetization curves acquired at the maximum of the Eu M_5_ edge for grazing (ϑ = 70°) and normal (ϑ = 0°) incidence geometries (T = 4 K). The arrows indicate the scanning direction.

X‐ray absorption spectroscopy (XAS) and x‐ray magnetic circular dichroism (XMCD) complement the ARPES observations, providing comparative insights into the magnetic properties of the intercalated systems. The XAS spectrum acquired in grazing incidence geometry at the Eu M_5,4_ edge for the graphene/Eu $\left(\sqrt{13}\times \sqrt{13}\right)R13.9^{\circ }$ /Re(0001) system features a sharp peak at the M_5_ edge along with a shoulder at higher photon energies. These contributions (marked by the black dashed lines in **Figure** **6**a) correspond to a mixed valence state of Eu(II) and Eu(III).^[^ ^37^ ^]^ The presence of the Eu(III) resonance may, in principle, be due to the formation of Eu_2_O_3_. However, this hypothesis can be ruled out based on the valence band data. Presuming the presence of the sesquioxide and considering the almost 3:1 O:Eu photoionization cross section at the selected photon energy, an intense O 2p contribution would be expected in the 4‐8 eV binding energy range,^[^ ^38^ ^][^ ^38^ ^]^ but no such feature is observed. On the other hand, the origin of the Eu(III) contribution may be attributed to variations of the Eu Wiegner‐Seitz (WS) radius. While bulk europium, characterized by a nearest neighbor distance of ≈3.99 Å (with a WS radius of 2.27 Å), possesses a pure (II) oxidation state, a decrease in the Eu WS radius leads to the emergence of a mixed Eu(II) and Eu(III) valence state.^[^ ^39^ ^]^ Indeed, decreasing the WS radius, the europium valence increases as electrons are squeezed out of the 4𝑓 shell into the 𝑠, 𝑝, 𝑑‐conduction band. For a WS radius of ≈2.01 Å, the (III) oxidation state is predicted to be nearly isoenergetic to the (II) one, a condition observed in the dense $\left(\sqrt{13}\times \sqrt{13}\right)R13.9^{\circ }$ phase. From the corresponding XAS spectrum (Figure 6a), a Eu(II)/Eu(III) ratio of 1:0.46 is found. Instead, when going toward the diluted (4 × 4) phase, the WS radius increases to ≈2.22 Å, a value close to the Eu bulk one, hence favoring the (II) oxidation state. This is consistent with the XAS spectrum acquired for the (4 × 4) superstructure (Figure 6b), which is characterized by the sole presence of the Eu(II) contribution.

Building on the understanding of the electronic structure, the magnetic properties of these intercalated systems were analyzed through absorption spectra acquired using right‐ and left‐circularly polarized light (µ_+_ and µ_‐_) in grazing and normal incidence geometries under varying magnetic fields. The absorption and the XMCD spectra for the two intercalated systems, acquired at normal incidence and with an applied magnetic field of 6 T at 4 K, are plotted in Figures 6c,d. The line shapes of the XMCD spectra shown in panels c and d reveal an overall Eu(II) character of the M_5,4_ edge, corresponding to an ^8^F_7/2_ ground state, displaying a strong ferromagnetic behavior, in line with the observations for intercalated Eu at the Gr/Ir(111) interface.^[^ ^33^ ^]^ Concurrently, according to Hund's rules, one would expect a nonmagnetic ^7^F_0_ ground state of Eu(III) (S = 3, L = 3, J = 0). Even though, due to the nonvanishing interaction with the external magnetic field as compared to the spin‐orbit interaction, there is a tiny magnetic moment in the 4f shell, Eu(III) XMCD is several orders of magnitude smaller compared to Eu(II). The magnetization arising from the van Vleck paramagnetism of Eu(III) due to the admixture of low‐lying excited states is also small, with a negligible contribution to the XMCD spectral shape. Indeed, in the case of the graphene/Eu $\left(\sqrt{13}\times \sqrt{13}\right)R13.9^{\circ }$ /Re(0001) system (Figure 6c), the XMCD spectrum shows no sizeable magnetic response at the Eu(III) position. The expectation values of the spin and orbital momenta have been accessed by applying sum‐rule analysis to the XAS‐XCMD spectra,^[^ ^40^, ^41^ ^]^ and are reported in Figure S4 (Supporting Information) of the Supporting Information. The results disclose the tendency to an isotropic behavior in both spin and orbital momenta for both phases, i.e., $m_{L}^{⊥}\approx m_{L}^{∥}$ and $\;m_{S}^{⊥}\approx m_{S}^{∥}$ within the experimental errors, as expected for the half‐filled 4f shell of Eu(II). It is worth noting that both structures do not present a quenched orbital moment, as one would expect for a half‐filled 4f shell, which remarks a significant hybridization between the graphene π‐band and the Eu 4f band and a strong spin‐orbit coupling imposed by the Re heavy metal. In the case of the graphene/Eu $\left(\sqrt{13}\times \sqrt{13}\right)R13.9^{\circ }$ /Re(0001) system, the magnetic moment is somewhat lower than the expected value of 7 µ_B_​. This phenomenon may be attributed to a non‐dichroic (paramagnetic or antiferromagnetic) contribution originating from Eu(III).^[^ ^42^ ^]^ Upon transitioning to the (4 × 4) phase, the XMCD response shows slight changes, with the spin moment increasing toward the expected value of 7 µ_B_ while preserving non‐negligible orbital moment​a. The magnetization curves (Figure 6e,f) reveal further details about the magnetic behavior. Both systems exhibit ferromagnetism and an in‐plane easy magnetization axis. The $\left(\sqrt{13}\times \sqrt{13}\right)R13.9^{\circ }$ phase shows a stronger ferromagnetic response, with a remanence magnetization corresponding to the 10% (35%) of the saturation value, in normal (grazing) incidence geometry. The corresponding spectra, acquired in remanence, are displayed in Supporting Information Figure S5 (Supporting Information). The observed ferromagnetic behavior arises from Eu‐Eu interactions enhanced by the Ruderman–Kittel–Kasuya–Yosida (RKKY) mechanism, mediated by the metallic substrate.^[^ ^33^ ^]^ The density and arrangement of Eu on the substrate play crucial roles in these interactions. As Eu coverage decreases during the structural transition to the (4 × 4)  phase, the ferromagnetic response weakens, resulting in a rounded hysteresis loop and suppressed magnetization remanence (1% in normal and 9% in grazing incidence). This highlights the critical influence of Eu coverage on the system's magnetic properties and underscores the interplay between interfacial structure and magnetism.

A sizable magnetic response is also observed at room temperature. As visible from the spectra acquired in grazing and normal incidence geometry (Figure S6, Supporting Information) both systems exhibit magnetic dichroism under an applied field of μ_0_ *H* = 6 *T*, with the magnetization reaching the value of ≈2%, suggesting a paramagnetic behavior at room temperature.

## 3 Conclusion

This study provides a systematic exploration of the phases formed by europium at the graphene/rhenium interface, demonstrating a direct correlation between structural ordering, electronic doping, and magnetism. The deposition and intercalation of europium result in distinct thermally stable phases, starting with the initial $\sqrt{3}\times \sqrt{3}R30^{\circ }$ reconstruction, which transforms into a $\left(\sqrt{13}\times \sqrt{13}\right)R13.9^{\circ }$ phase upon intercalation and eventually transitions to a diluted (4 × 4) phase at higher annealing temperatures, underscoring the crucial role of the rhenium substrate in modulating the europium density. The rare earth intercalation significantly modifies graphene's electronic properties, shifting the Dirac point by more than 1.5 eV from the Fermi level. In both Eu superstructures, the majority branch of the Dirac cone hybridizes with Eu 4f states and is displaced in energy, while the minority one remains largely intact and preserves a Dirac‐like dispersion. The charge transfer, in the europium dense phase, is sufficient to occupy the π* band, leading to the presence of flat bands at the $\bar{M}$ point of the SBZ. X‐ray absorption spectroscopy and x‐ray magnetic circular dichroism demonstrate ferromagnetism primarily driven by Eu(II), with a transition from a mixed Eu(II)/Eu(III) valence state in the europium dense phase to a pure Eu(II) state in the diluted phase. A persistent magnetic response is observed even at room temperature.

The ability to precisely tune the charge transfer, bandgap, and magnetic response highlights europium‐intercalated graphene as a promising platform for next‐generation quantum materials and reconfigurable electronic devices. These results pave the way for further exploration of rare‐earth intercalation in 2D materials with exceptional thermal stability, expanding the possibilities for functional interfaces in nanoelectronics and spintronics.

## 4 Experimental Section

### Sample Preparation

Re(0001) was used as a substrate for all the experiments. The sample was cleaned by oxygen treatment (p = 3 × 10^−7^ mbar; T = 1070 K) followed by high‐temperature flashes to above 1850 K in ultra‐high vacuum (UHV). Graphene was grown using chemical vapor deposition (CVD). The clean rhenium surface was initially exposed to ethylene (C_2_H_4_) at room temperature, and subsequently, the substrate underwent annealing cycles between 570 and 970 K, following established growth protocols.^[^ ^31^ ^]^ Europium was inserted into a homemade crucible and thoroughly outgassed for about one week. The deposition was performed on the Gr/Re(0001) surface, as detailed in the results section, with a rate of ≈0.65 Å min^−1^ while keeping the surface at 470 K to avoid contamination of the Eu overlayer, while still not promoting intercalation.

### Experimental Methods

The experiments were performed at the Nanospectroscopy endstation of the Elettra Synchrotron in Trieste (Italy) and at the Boreas beamline of the ALBA synchrotron in Barcelona (Spain).

The LEEM and LEED, as well as ARPES experiments, were carried out at the Nanospectroscopy beamline using the Elmitec Spectroscopic Photoemission and Low Energy Electron Microscope (SPELEEM).^[^ ^43^, ^44^, ^45^ ^]^ The microscope can operate in real space (LEEM) and reciprocal space (LEED) modes. In this instrument, the electron energy was chosen by applying a voltage bias to the sample, commonly known as the start voltage. LEEM allows one to image the specimen surface at video frame rate and hence can be employed for real‐time imaging of dynamical processes at surfaces (with lateral resolution better than 10 nm). When coupled with an X‐ray source with variable energy and polarization, the SPELEEM allows for imaging the chemical and magnetic properties of the sample (XPEEM and XMCD‐PEEM) when operating in real space mode and for imaging the band structure (ARPES) when operating in reciprocal space mode. In this work, all the ARPES measurements were performed using 40 eV photons with vertical polarization (p‐pol).

XAS and XMCD spectra were acquired at the BOREAS beamline.^[^ ^46^ ^]^ The applied magnetic field and temperature for acquiring the XMCD data were B = 6 T and T ≈4 K. Measurements were taken at the Eu M_4,5_ edges, with the magnetic field fixed in the direction of the incident light. The spectra were collected by the total electron yield mode. Angle‐dependent measurements were carried out by rotating the sample about a vertical axis perpendicular to the synchrotron orbital plane, thereby varying the incidence angle between the X‐ray beam (and, therefore, the magnetic field) and the substrate normal.

### Theoretical Calculations

DFT was employed in the generalized gradient approximation^[^ ^47^ ^]^ using van der Waals corrections in the D3 form.^[^ ^48^ ^]^ The calculations were performed with the full‐potential linearized augmented planewave method in thin‐film geometry^[^ ^49^ ^]^ as implemented in the Fleur code.^[^ ^50^ ^]^ To describe the Eu 4f electrons, a Hubbard U of 5.9 eV and J = 0.9 eV was used as in a previous study.^[^ ^12^ ^]^ Graphene was placed in a (1x1) register with the underlying Re(0001) substrate in a hcp‐top adsorption geometry where the Graphene lattice constant was maintained. The intercalated Eu was placed on top of three Re(0001) layers. The structural relaxation was performed by allowing the Eu and C atoms to freely move, while keeping the underlying Re layers in a fixed geometry. Such a constrained geometry may slightly influence the DFT‐derived Fermi level. For reaching self‐consistency, a 3 × 3 × 1 k‐point set has been used. The magnetic order was assumed to be ferromagnetic. The band unfolding was performed as described in Ref.[51]

## Conflict of Interest

The authors declare no conflict of interest.

## Author Contributions

M.J., I.C., and T.O.M. contributed equally to this work. The manuscript was written through the contributions of all authors. All authors have given approval to the final version of the manuscript. ‡M.J., and I.C. contributed equally. M.J., I.C., and P.P. conceptualized the project, led the investigation and funding acquisition, with support from A.L. G.B. performed the DFT calculations with support from S. Blügel. M.J. and I.C. performed the experiments and data analysis, wrote the original manuscript with support from all the authors.

## Supporting information



Supporting Information

## Data Availability

The data that support the findings of this study are available from the corresponding author upon reasonable request.
