# Supplementary material for: Persistent Magnetism and Tunable Doping of Monolayer Graphene via Europium Density Modulation
Source: Adv Sci (Weinh). 2025 Nov 21;13(7):e21592. doi: 10.1002/advs.202521592 (PMC12866880; doi:10.1002/advs.202521592)
Supplement: Supplementary file 1 — Supporting Information [file ADVS-13-e21592-s001.docx]

Supporting Information

Persistent magnetism and tunable doping of monolayer graphene via europium density modulation

M. Jugovac^§,1,2^*, I. Cojocariu^§,1,2^, G. Bihlmayer^3^, P. Gargiani^4^, M. S. Valvidares^4^, C. A. Brondin^2,5^, S. Blügel^3,6^, A. Locatelli^2^, T. O. Menteş^2^ and P. Perna^7^*

^1^ Dipartimento di Fisica, Università degli studi di Trieste, Via A. Valerio 2, 34127 Trieste, Italy
^2^ Elettra Sincrotrone Trieste S.C.p.A., Strada statale 14, km 163.5, 34149 Trieste, Italy

^3^ Peter Grünberg Institut (PGI-1), Forschungszentrum Jülich and JARA, 52425, Jülich, Germany

^4^ ALBA Synchrotron Light Source, Carrer de la Llum 2-26, 08290 Barcelona, Spain
^5^ CNR-Istituto di Struttura della Materia (CNR-ISM), Strada Statale 14, km 163.5, 34149 Trieste, Italy

^6^ Institute for Theoretical Physics, RWTH Aachen University, 52056 Aachen, Germany
^7^ IMDEA Nanociencia, C/Faraday 9, Campus de Cantoblanco, 28049 Madrid, Spain
^§^ These authors contributed equally to this work

Correspondence: [matteo.jugovac@units.it](mailto:matteo.jugovac@units.it) ; [paolo.perna@imdea.org](mailto:paolo.perna@imdea.org)

^‡^ These authors contributed equally to this work


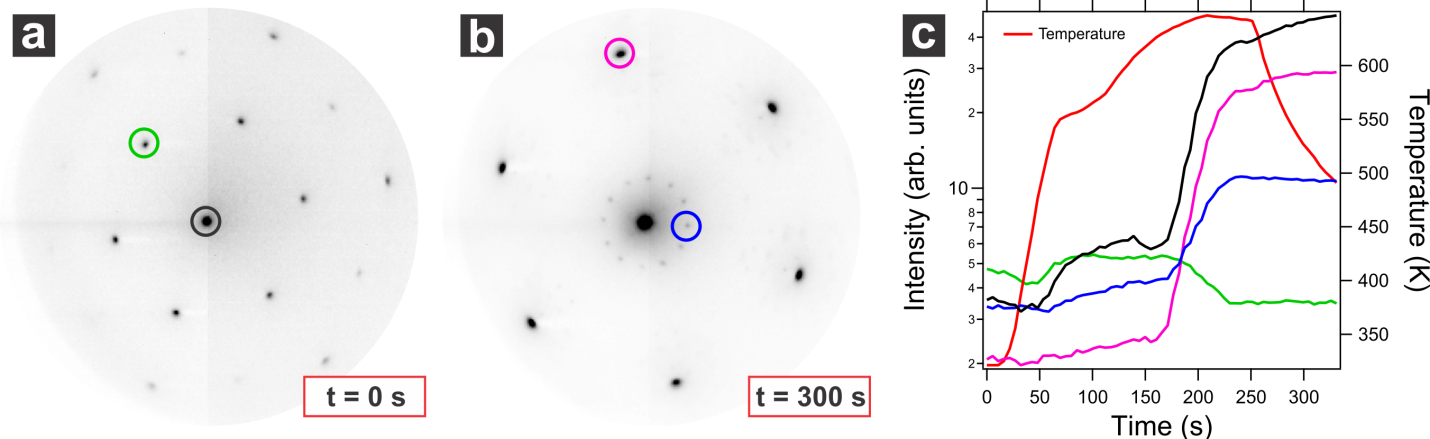


*Figure S1. a) LEED pattern (E_k_ = 60 eV) of the Eu-induced* $\sqrt{\boldsymbol{3}}\boldsymbol{\times}\sqrt{\boldsymbol{3}}\boldsymbol{R}\boldsymbol{30^{\circ}}$ *reconstruction on Gr/Re(0001), recorded after the deposition of metallic Eu at 470 K. b) LEED pattern (E_k_ = 60 eV) after Eu intercalation at the Gr/Re(0001) interface upon annealing to 650 K. c) LEED intensity profile vs. time of the spots marked in a) and b).*


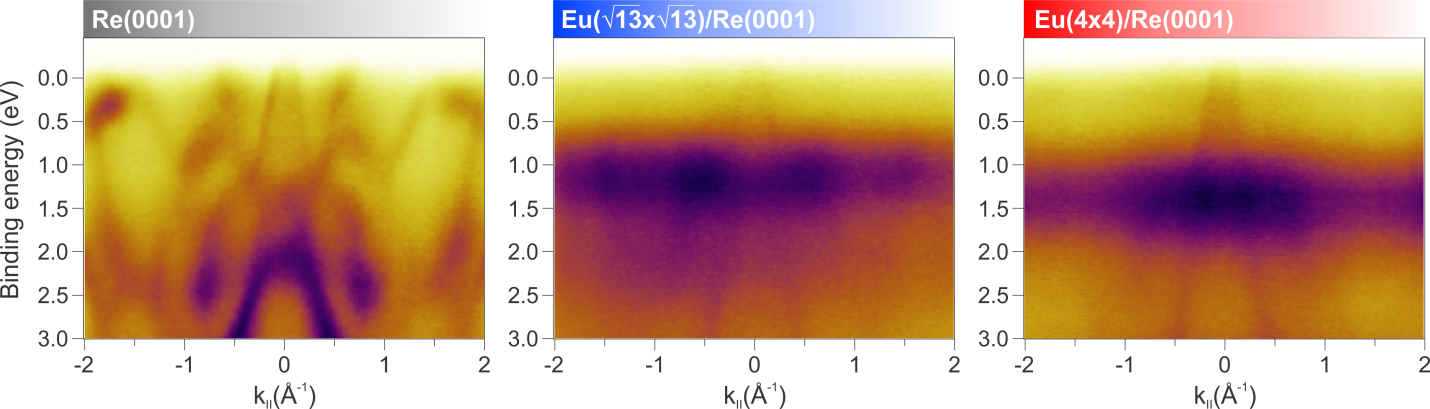


*Figure S2. Energy vs. momentum maps acquired along the* $\overline{\boldsymbol{K\Gamma K}}$ *direction of: a) clean Re(0001), b) Eu*$\boldsymbol{(}\sqrt{\boldsymbol{13}}\boldsymbol{\times}\sqrt{\boldsymbol{13}}\boldsymbol{)R}\boldsymbol{13.9^{\circ}}$*/Re(0001) and c) Eu*$\boldsymbol{(4\times4)}$*/Re(0001). Photon energy 40 eV, p-pol.*

*
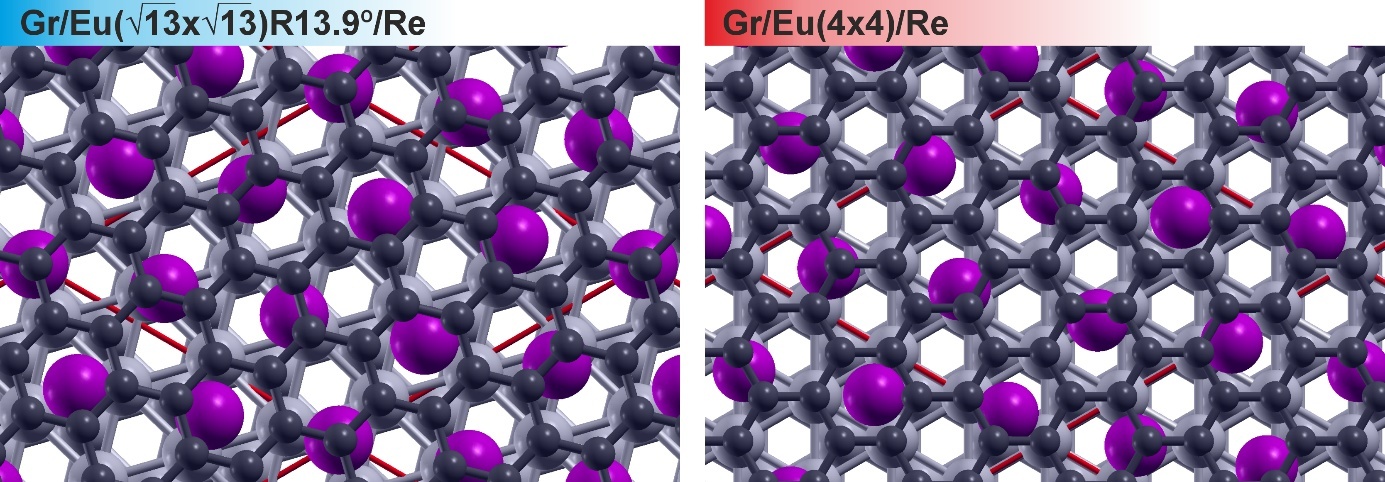
*

*Figure S3. Structurally optimized DFT unit cells used for the calculation of the band maps displayed in the main text. Re, Eu and C atoms are marked by gray, purple and black spheres, respectively.* *Flipping the magnetization of a single Eu atom of the denser structure resulted in an higher energy of 24.3 meV per Eu pair, therefore the magnetic order was assumed to be ferromagnetic.*

*
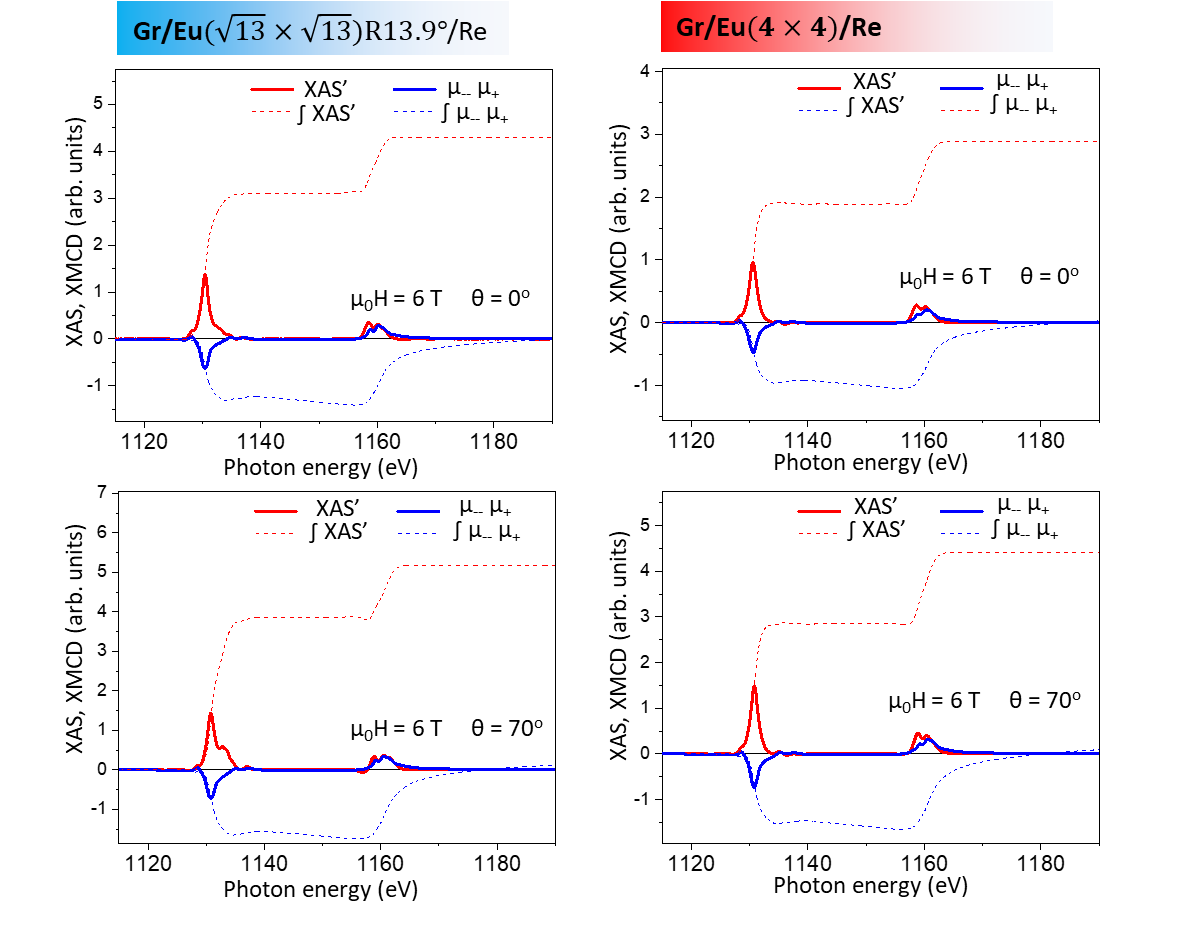
*

*Figure S4. XAS-XMCD spectra acquired in normal and grazing incidence geometry for the two structural phases at T = 4 K (μ_0_H = 6 T) and corresponding integrals for the application of sum rules. XAS’ is the background corrected XAS.*


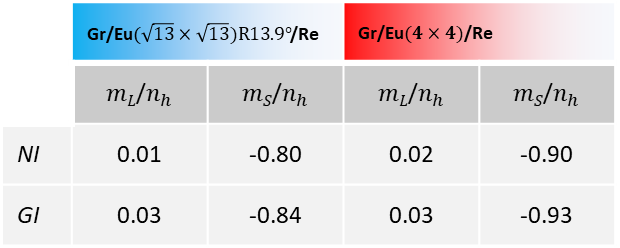


Table S1. Results of sum rules applied to the spectra in Figure S3.


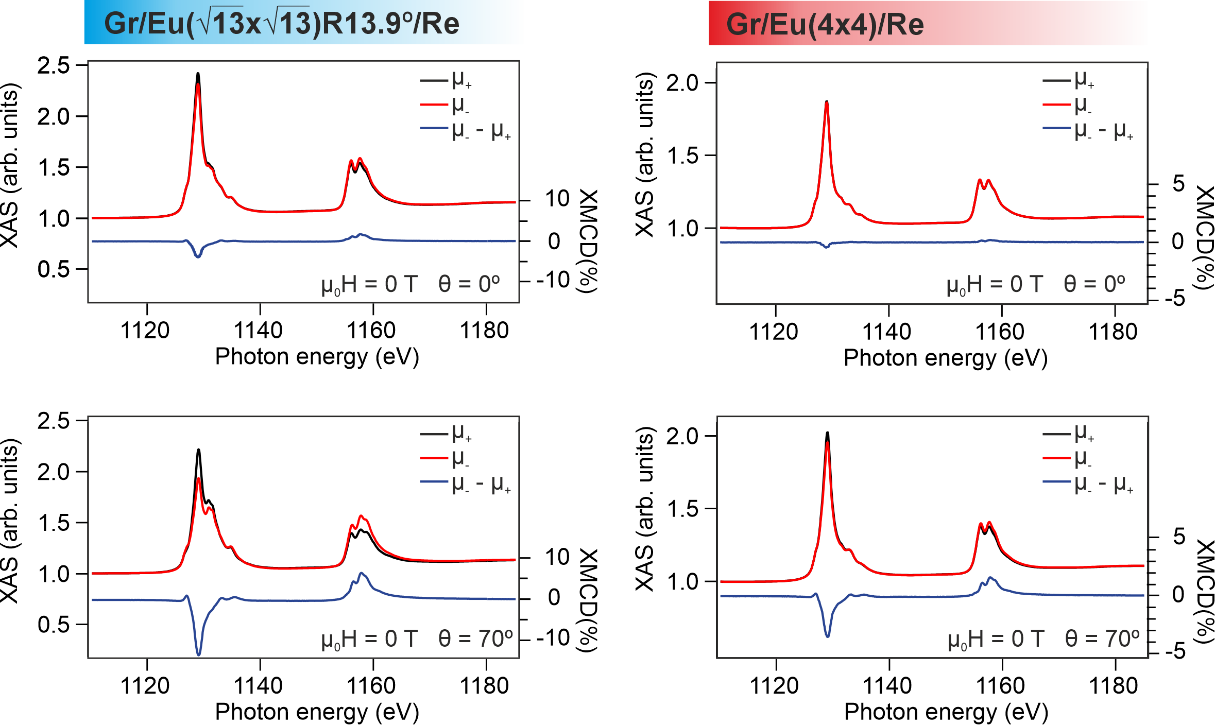


*Figure S5. XAS-XMCD spectra acquired in normal and grazing incidence geometry for the two structural phases in remanence (T = 4 K).*


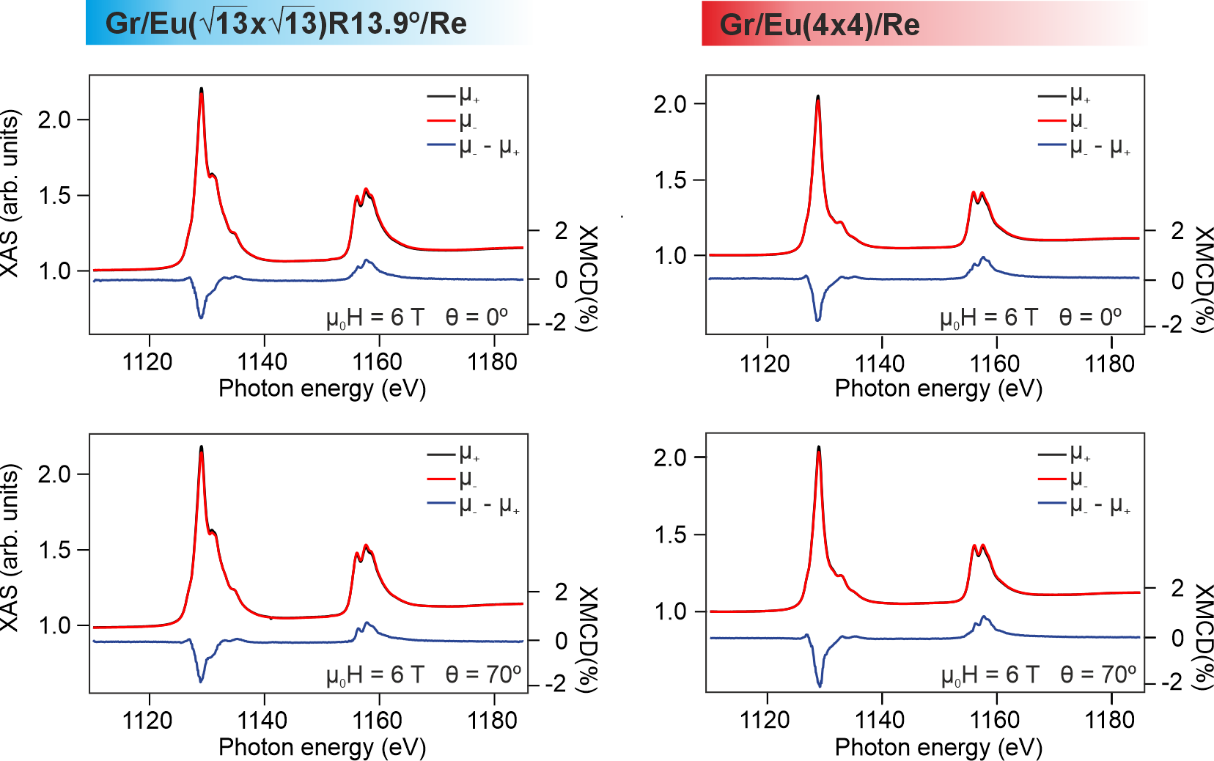


*Figure S6. XAS-XMCD spectra acquired in normal and grazing incidence geometry for the two structural phases at T = 300 K (μ_0_H = 6 T).*
